# Treatment with lipoxin A 4 improves influenza A infection outcome through macrophage reprogramming, anti-inflammatory and pro-resolutive responses

**DOI:** 10.21203/rs.3.rs-4491036/v1

**Published:** 2024-06-13

**Authors:** Flavia Rago, Eliza Mathias Melo, Leigh M. Miller, Alexis M. Duray, Franciel Batista Felix, Juliana Priscila Vago, Ana Paula Faria Gonçalves, Ana Luiza Pessoa Mendonça Angelo, Giovanni D. Cassali, Monica Gaetano, Eoin Brennan, Benjamin Owen, Patrick Guiry, Catherine Godson, John F. Alcorn, Mauro Martins Teixeira

**Affiliations:** UMPC Children’s Hospital of Pittsburgh; Universidade Federal de Minas Gerais; UMPC Children’s Hospital of Pittsburgh; UMPC Children’s Hospital of Pittsburgh; Universidade Federal de Minas Gerais; Universidade Federal de Minas Gerais; Oswaldo Cruz Foundation (FIOCRUZ-Minas); Oswaldo Cruz Foundation (FIOCRUZ-Minas); Universidade Federal de Minas Gerais; University College Dublin; University College Dublin; University College Dublin; University College Dublin; University College Dublin; UMPC Children’s Hospital of Pittsburgh; Universidade Federal de Minas Gerais

**Keywords:** Influenza A, Inflammation, Pneumonia, Respiratory Infection, Resolution, Pro-resolving mediators

## Abstract

**Objective and design:**

Here, we evaluated whether a synthetic lipoxin mimetic, designated AT-01-KG, would improve the course of influenza A infection in a murine model.

**Treatment:**

Mice were infected with influenza A/H1N1 and treated with AT-01-KG (1.7 mg/kg/day, i.p.) at day 3 post-infection.

**Methods:**

Mortality rate was assessed up to day 21 and inflammatory parameters were assessed at days 5 and 7.

**Results:**

AT-01-KG attenuated mortality, reducing leukocyte infiltration and lung damage at day 5 and day 7 post-infection. AT-01-KG is a Formyl Peptide Receptor 2 (designated FPR2/3 in mice) agonist, and the protective responses were not observed in FPR2/3 ^−/−^ animals. In mice treated with LXA_4_ (50mg/kg/day, i.p., days 3–6 post-infection), at day 7, macrophage reprogramming was observed, as seen by a decrease in classically activated macrophages and an increase in alternatively activated macrophages in the lungs. Furthermore, the number of apoptotic cells and cells undergoing efferocytosis was increased in the lavage of treated mice. Treatment also modulated the adaptive immune response, increasing the number of anti-inflammatory T cells (Th2) and regulatory T (Tregs) cells in the lungs of the treated mice.

**Conclusions:**

Therefore, treatment with a lipoxin A_4_ analog was beneficial in a model of influenza A infection in mice. The drug decreased inflammation and promoted resolution and beneficial immune responses, suggesting it may be useful in patients with severe influenza.

## 1. INTRODUCTION

Influenza A virus (IAV) is a virus from the Orthomyxoviridae family responsible for high levels of mortality and morbidity worldwide^1^ and the leading pathogen of many outbreaks and pandemics such as Spanish Flu (1918), Asian Flu (1957), Hong Kong Flu (1968) and Swine Flu (2009)^2,3^. The World Health Organization estimates one billion cases of IAV infection per year with 3–5 million severe cases and 300.000–500.000 deaths^4^. Although there are vaccines and anti-viral drugs used for the treatment of influenza infection^5^, due to the high level of mortality and morbidity, finding additional strategies to minimize the impact of this infection on public health is greatly necessary.

Influenza virus infects the upper and lower respiratory tract and can reach the alveolar epithelium and alveolar macrophages^6^. Binding between the sialic acid on the surface of host cells and viral hemagglutinin^7^ triggers the death of infected cells by apoptosis and necroptosis^8,9^. The activation of apoptosis of infected cells is important to control viral replication and, consequently, decreases viral spread specially when the virus escapes the immune system^10^. Once infected, cells recognize pathogen associated molecules patterns (PAMPs) through the activation of toll like receptors (TLRs) inducing not only the recruitment of immune cells to the site of infection, but also the production of cytokines such as interleukin (IL)-1β, IL-6 and interferons (IFNs)^10–13^. Macrophages and neutrophils are the most recruited cells into the infected lungs during IAV infection^14,15^. In the beginning of infection, macrophages are classically activated expressing markers such as iNOS and MHCII, along with cytokines and neutrophil extracellular traps (NETs), they are crucial to prevent viral replication^16,17^.

Host mediated immune pathology is a critical contributor to lung injury during IAV infection. A better outcome of infection relies on the activation of the resolution phase of inflammation in which anti-inflammatory cytokines and pro-resolving mediators are produced^18^. We have previously shown that treatment with certain pro-resolving molecules is beneficial in the course of viral infections, including dengue virus, chikungunya virus and IAV^19–21^. For example, treatment with annexin-A1 greatly prevented disease severity after dengue virus infection and shortened chronicity in a model of chikungunya virus infection^20^. Similarly, treatment with angiotensin 1–7 (Angio 1–7), a pro-resolving molecule, significantly decreased pulmonary inflammation and damage associated with IAV infection in mice, an effect associated with fewer animals succumbing to infection^21^. These studies have led to the suggestion that pro-resolving molecules may be an useful adjunct therapy for the treatment of infectious diseases^22^.

Lipoxin A_4_ (LXA_4_) is an eicosanoid that promotes the resolution of inflammation through pleiotropic responses, are mediated via Formyl Peptide Receptor 2 (FPR2), a GPCR (designated FPR2/3 in mice). Chemical instability and complex synthesis have limited exploitation of the therapeutic potential of the lipoxins. We have developed several synthetic lipoxins mimetics where the triene core of LXA_4_ has been replaced by heteroaromatic moieties with enhanced chemical stability, such as AT-01-KG, which contains a dimethylimidazole core^23,24^. The LXA_4_ analogue AT-01-KG, a dimethyl-imidazole-containing lipoxin A_4_ analogue, has both classic anti-inflammatory effects by reducing neutrophil influx and pro-inflammatory cytokines but also pro-resolving effects^25^. In the current study, we evaluated whether treatment with AT-01-KG would favorably modify the course of IAV infection in mice. Mechanisms associated with protection were also evaluated.

## 2. MATERIAL AND METHODS

### 2.1. Mice

Male wild-type and FPR2/3^−/−^ (KO) C57BL/6J mice (8–12 weeks old), weighing 18–25g, were obtained from the Central Animal Facility from Universidade Federal de Minas Gerais (UFMG/Brazil) and from the Animal Facility from Instituto de Ciências Biológicas in UFMG/Brazil, respectively. Mice were randomized by sex and assigned to experimental groups. They were maintained with free access to commercial chow and water in a 12-h dark–light cycle in the thermoneutral zone for mice. All procedures described had prior approval of the local animal ethics committee (CETEA/ UFMG 65/2021). WT C57BL/6 (6 to 8-week-old) mice were also purchased from Jackson Laboratory (Bar Harbor, Maine) and colonies were subsequently maintained under specific pathogen-free conditions at UPMC Children’s Hospital of Pittsburgh. All animals’ procedures described here had prior approval of the animal ethics committee of the Federal University of Minas Gerais and the University of Pittsburgh Institutional Animal Care and Use Committee.

### 2.2. IAV infection

The influenza strains used in the study were influenza A WSN/33 H1N1 produced in chicken eggs and then co-cultured with MDCK cells as previous described^26^ and influenza A/PR/8/34 (influenza H1N1) propagated in chicken eggs as described^27^. Animals were anesthetized with ketamine/xylazine and received 10^4^ PFU (lethal dose 50%) as previous described^3^.

### 2.3. AT-01-KG and Lipoxin A_4_ treatments

**Figure 1:**
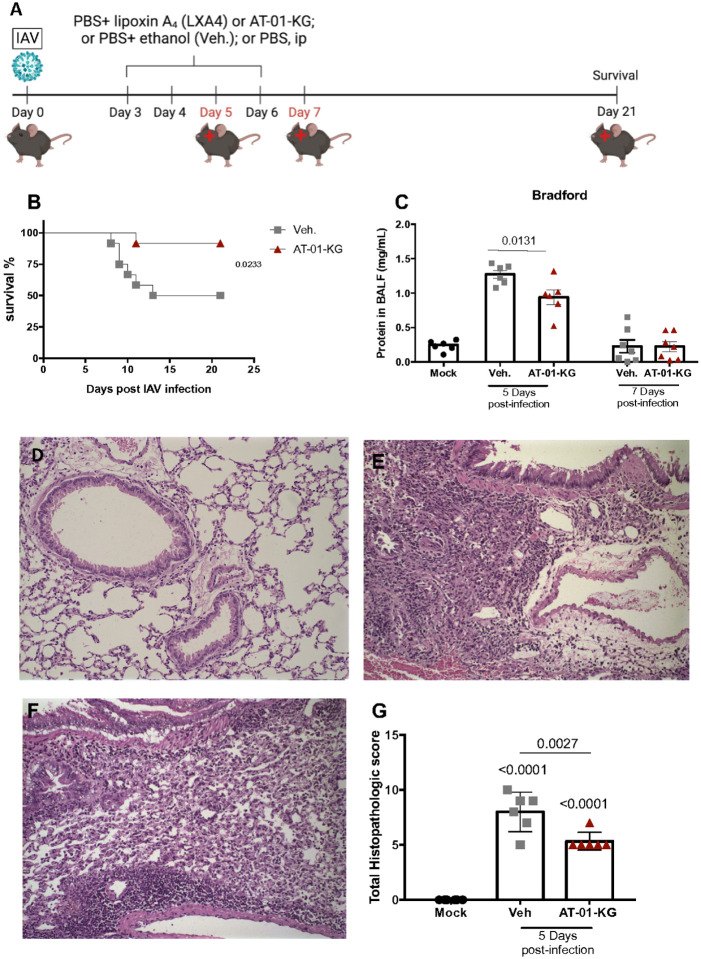
Treatment with AT-01-KG improves survival and tissue damage. WT (C57Bl/6) mice were infected with Influenza A H1N1 and euthanized 5 days and 7 days post infection. Mice were daily treated with vehicle (Veh.) or with lipoxin A_4_ analogous (AT-01-KG) from day 3 up to the day of euthanasia and a survival curve was also performed up to day 21 post-infection (A). Survival (B), bradford (C), lung histology from mock mice, vehicle-treated mice and lipoxin A4-treated mice (D, E and F, respectively) and histopathologic score were performed (G). N = 6. Results are representative of two independent experiments. Statistical analysis was done using area under the curve or One-way ANOVA, Turkey’s multiple comparisons test. P values were shown.

AT-01-KG was generated as previously described^24,28^. LXA_4_ was also purchased from Cayman Chemical Company (item: 90410; Purity ≥95%). Infection with IAV, sham infection and treatments with AT-01-KG (1.7 μg/kg/day, i.p.) or with LXA_4_ (50μg/kg/day, i.p.) and appropriate vehicles are depicted in Fig. 1A. Treatments started at 3 days post-infection and euthanized at days 5 and 7 or maintained up to 21 days post initial infection^29,30^

### 2.4. Assessment of lung Inflammation

The trachea was exposed, and bronchoalveolar lavage (BAL) was performed with two aliquots containing 1mL of sterile PBS. Saline solution was injected and collected three times into the lungs using a cannula and syringe. Then the BAL fluid (BALF) samples were centrifuged at 600 × g for 10 min at 4°C to count leukocytes. In addition, cytospin (Shandon III) of these leukocytes was performed for differential counting on slides stained with May-Grunwald-Giemsa, based on morphological criteria. Each slide was counted three times and the percentage was used to calculate the absolute number of each type of leukocyte, as previously described^31,32^. The right upper lobe of the lungs was mechanically homogenized in sterile PBS in order to measure cytokine production by Bio-plex assay (Bio-Rad, Hercules, CA) and the right middle and lower lobes were used for flow cytometry.

### 2.5. Assessment of Bone Marrow-Derived Macrophage (BMDM) efferocytosis

Bone marrow cells were sterilely isolated from the femurs and tibias of mice, and grown for 7 days in complete DMEM media supplemented with 20ng/mL GM-CSF (PeproTech, NJ), as previously described^33^. On day 6, adherent cells (BMDMs) were recovered using a cell scraper (Genemate, Kaysville, UT) with gentle mechanical scraping. Bone marrow derived cells were plated to 2.5 × 10^5^ cells/mL in 300ul of media in 96 well tissue culture-treated plates then rested overnight in complete DMEM media supplemented with 20ng/mL GM-CSF (PeproTech, NJ). BMDMs were infected with 0.1 MOI of influenza A WSN/33 H1N1 for 30min. Then, wells were washed with PBS 1% and complete DMEM was added with or without 1nM of AT-01-KG for 24 hours. On the following day CFSE labeled apoptotic thymocytes were generated as described^34^ and co-cultured with BMDMs for 1 hour at 37°C, at a ratio of 1:3. Finally, cells were superficially stained by labeled anti-F4/80 (PE-Cy7-eBioscience). Assessment of efferocytosis was performed by flow cytometry for F4/80^+^/CFSE^+^ cells.

### 2.6. Assessment of Leukocyte Apoptosis and Efferocytosis

Apoptosis and efferocytosis was assessed morphologically, as previously described^26,35^. BALF was collected 7 days post IAV infection. Cells were centrifuged, fixed, and stained with May-Grünwald-Giemsa and counted using oil immersion microscopy (100 objective) to determine the proportion of cells with distinctive apoptotic morphology (cells presenting chromatin condensation, nuclear fragmentation, and formation of apoptotic bodies, out or inside macrophages) and macrophage undergoing efferocytosis. At least 300 cells were counted/slide. Efferocytosis was also assessed by flow cytometry considering cells F480+/CSFE+.

### 2.7. Flow Cytometry analysis

For *in vivo* experiments, mouse lungs were aseptically dissected into small sections, digested for 1 hour at 37°C in 1mg/mL collagenase media, and filtered with 70μm filters^36^. Two panels were made for myeloid and lymphoid cell staining. After live/dead staining with LIVE/DEAD blue or Zombie NIR, extracellular staining was done at 4°C for 30 minutes. Single cell suspensions staining is shown (Supplementary Fig. 4). The gating strategy used with FlowJo (Tree Star, Ashland, OR) is shown (Supplementary Figs. 2 and 3). Dimensionality reduction t-SNE analysis followed by FlowSOM clustering analysis was performed using Cytobank (Beckman Coulter, Indianapolis, IN). For in vitro experiments, cells from BAL were stained by labeled antibodies anti-F4/80 (25–4801- 82, PEcy7, eBioscience, USA).

### 2.8. Histopathology assessment

After the reperfusion of the lungs, the left lung was collected and fixed in 4% neutral phosphate-buffered formalin (pH 7.4), as described previously^37^. Tissues were dehydrated gradually in ethanol, embedded in paraffin, cut into 4mm sections, stained with hematoxylin-eosin, and analyzed under light microscopy by a pathologist blinded to the treatments. Lung structure, inflammation, edema and hemorrhage were quantified using a semi-quantitative score with increasing severity of changes (0, absent; 1, minimal; 2, slight; 3, moderate; 4, marked; and 5, severe). All parameters were included in an overall 20 points score.

### 2.9. Statistical Analysis

Data were analyzed using GraphPad Prism (Boston, MA) software. Experiments were repeated twice. All data are presented as mean ± SEM. Significance was determined by unpaired Student’s t test or one-way ANOVA followed by post-hoc Bonferroni or Tukey’s test for multiple comparisons. Area under the curve were used for mortality data analysis followed by a one-way ANOVA test.

## 3. Results

### 3.1. Treatment with AT-01-KG improved survival, lung damage and reduced cell infiltration

LXA_4_ is a specialized pro-resolving mediator that contributes to control of inflammation, reducing lung damage^38^. AT-01-KG is a synthetic lipoxin-A4 mimetic where the triene core of LXA_4_ is replaced by a heteroaromatic imidazole. In order to analyze the potential effect of AT-01-KG as a potential treatment for IAV infection, we analyzed three distinct groups: PBS-treated uninfected mice (Mock), vehicle treated IAV infected mice (Veh.) and AT-01-KG treated IAV infected mice (AT-01-KG). Daily treatment was performed intraperitonially (ip) starting on day 3 post-infection up to the day of evaluation. Vehicle and AT-01-KG groups were infected with H1N1 IAV and inflammatory parameters were evaluated 5 and 7 days post-infection along with a survival curve (Fig. 1A). Mice treated with AT-01-KG showed improved survival (Fig. 1B). As anticipated, IAV infection was associated with increased protein in BALF (Fig. 1C). The increase protein in BALF was significantly reduced in AT-01-KG treated mice 5 days post infection relative to vehicle treated mice. Histopathology analysis showed increased inflammation and damage in the lungs of untreated mice compared to the mock group (Figs. 1D and E). However, decreased inflammation and damage were seen in the lungs of treated mice compared to untreated mice (Figs. 1F and G).

**Figure 2:**
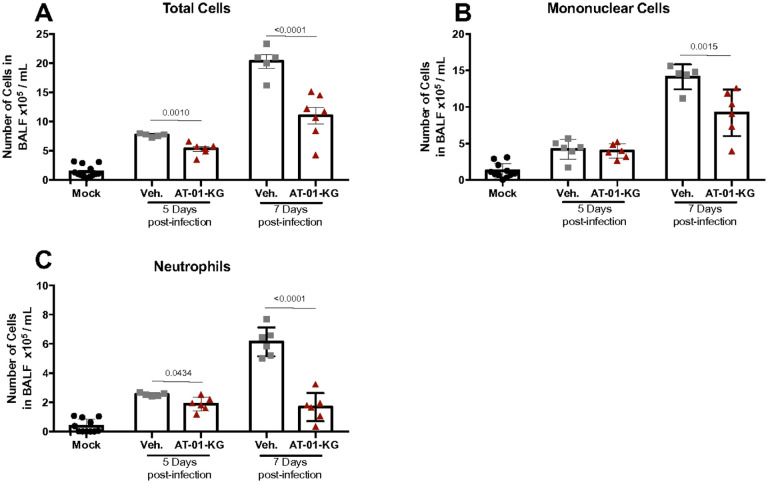
AT-01-KG treatment decreases number of cells in BALF. WT (C57Bl/6) mice were infected with Influenza A H1N1 and euthanized 5 days and 7 days post infection. Mice were daily treated with vehicle (Veh.) or with lipoxin A_4_ analogous (AT-01-KG) from day 3 up to the day of euthanasia. Total cells (A), Mononuclear cells (B) and Neutrophils (C) were counted based on morphology. N = 6. Results are representative of two independent experiments. Statistical analysis was done using One-way ANOVA, Turkey’s multiple comparisons test. P values were shown.

Even though the treatment did not affect the ability of the host to control viral replication (Supplementary Fig. 1A), BALF analysis showed reduced cell infiltration induced by the AT-01-KG treatment (Fig. 2A), particularly reduced numbers of mononuclear cells (Fig. 2B) and neutrophils (Fig. 2C) on day 7 post-infection. Thus, we decided to further investigate the pathology 7 post-infection in vehicle-treated and LXA4-treated mice. As anticipated, AT-01-KG treatment did not impact the number of cells in the mock group (Supplementary Fig. 1B).

### 3.2. LXA_4_ treatment induced macrophage reprogramming towards an alternatively activated phenotype

**Figure 3:**
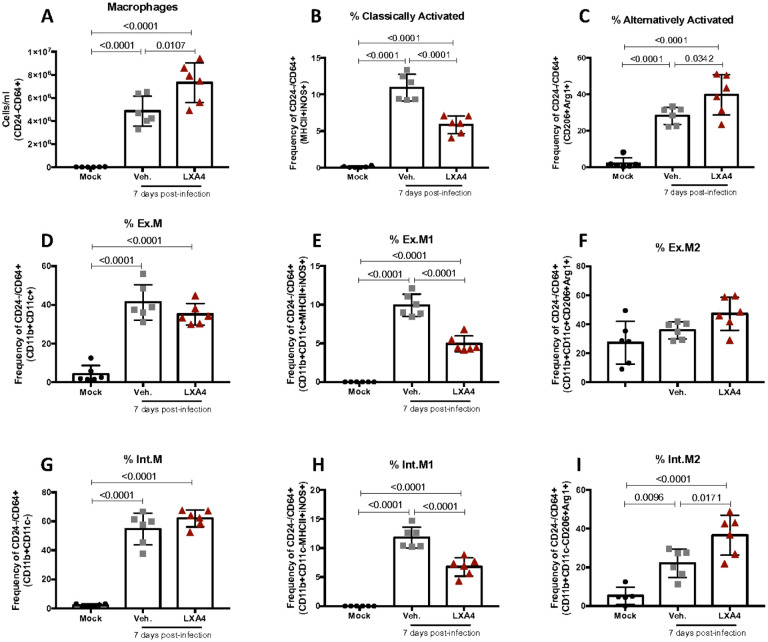
Lipoxin A_4_ treatment induces macrophage polarization. WT (C57Bl/6) mice were infected with Influenza A H1N1 and euthanized 7 days post infection. Mice were daily treated with vehicle (Veh.) or with lipoxin A4 (LXA4) from day 3 up to the day 6. Flow cytometry of Macrophages (CD24-CD64+ )(A), classically activated macrophages (CD24-CD64+MHII+iNOS)(B), alternatively activated macrophages (CD24-CD64+CD206+arg1+)(C) Exudate Macrophages (Ex.M – CD24-CD64+CD11b+CD11c+)(D), classically activated exudate macrophages (Ex. M1 – CD24-CD64+CD11b+CD11c+MHCII+iNOS+)(E), alternatively activated exudate macrophages (Ex. M2 – CD24-CD64+CD11b+CD11c+CD206+arg1+)(F), Interstitial Macrophages (Int.M – CD24-CD64+CD11b+CD11c-)(G), classically activated interstitial macrophages (Int.M1 – CD24-CD64+CD11b+CD11c-MHCII+iNOS+)(H), alternatively activated interstitial macrophages (Int.M2 – CD24-CD64+CD11b+CD11c-CD206+arg1+). N= 6. Results are representative of two independent experiments. Statistical analysis was done using One-way ANOVA, Turkey’s multiple comparisons test. P values were shown.

Macrophage reprogramming from a classically activated phenotype towards an alternatively activated phenotype is a crucial step in the resolution phase of inflammation to control pro-inflammatory response contributing to an anti-inflammatory and pro-resolutive environment^39^. Specialized pro-resolving mediators, such as LXA_4_, are known to be able to induce macrophage reprogramming during resolution^38^. Flow cytometry analysis showed that LXA_4_ treatment not only increased the number of macrophages in the lungs of treated mice (Fig. 3A and Supplementary Fig. 2) but also decreased frequency of macrophages expressing classically activated markers such as iNOS and MHCII (Fig. 3B). The treatment was also associate with increased frequency of macrophages expressing alternatively activated markers such as Arg1 and CD206 (Fig. 3C). Macrophages subpopulation analysis showed that infected mice presented increased frequency of exudate macrophages (CD24-CD64 + CD11b + CD11c+) (Fig. 3D) and interstitial macrophages (CD24-CD64 + CD11b + CD11c-) (Fig. 3G), however, reduced the frequency of alveolar macrophages (Supplementary Fig. 1C). The treatment reduced the frequency of exudate and interstitial macrophages expressing classically activated markers (Figs. 3E and H) and increased interstitial macrophages expressing alternatively activated markers (Figure I). There was a trend towards an increase in the expression of alternatively activated markers in exudate macrophages (Fig. 3F). Alveolar macrophages from infected mice did not show significantly increased expression of iNOS, MHCII, Arg1 and CD206 following IAV infection preventing the analysis of macrophage reprogramming in this population (data not shown).

### 3.3. Frequency of apoptotic cells and macrophage efferocytosis was increased by AT-01-KG treatment

**Figure 4:**
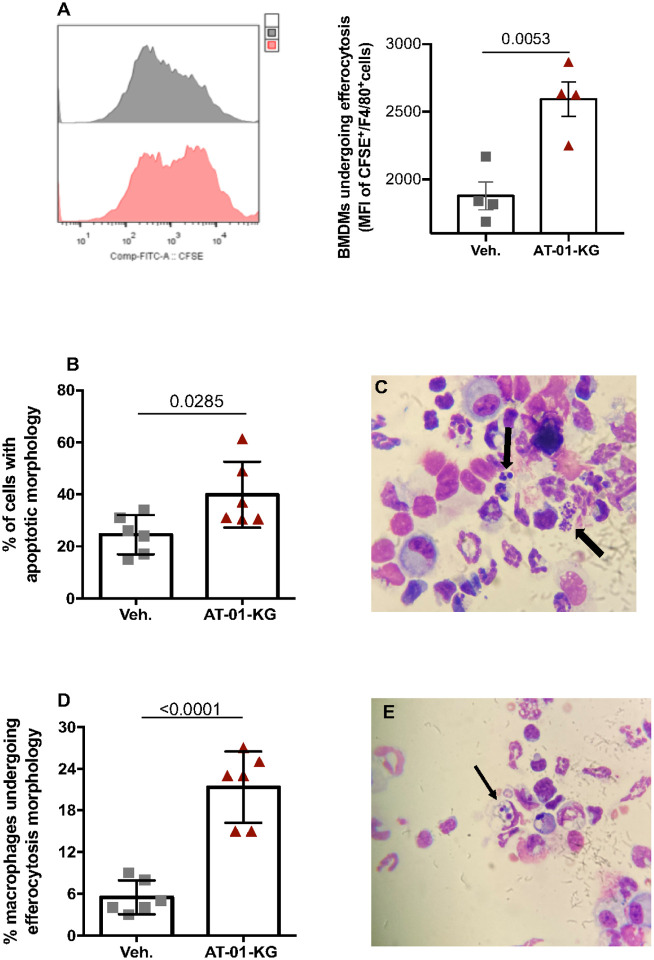
AT-01-KG treatment Increases apoptosis and efferocytosis *in vitro* and *in vivo*. Bone marrow-derived macrophages were differentiated in 6 well/plate. The cells were treated with 1nM of lipoxin A_4_ analogous (AT-01-KG) for 24 hours then infected with an MOI 0.1 of Influenza A HIN1 (FLU) for 30 minutes. Apoptotic cells were added to each well for 1 hour. Macrophages undergoing efferocytosis (F480+CFSE+) were analyzed by flow cytometry (A). WT (C57Bl/6) mice were infected with Influenza A H1N1 and euthanized 7 days post infection. Mice were daily treated with vehicle (Veh.) or with lipoxin A_4_ analogous (AT-01-KG) from day 3 up to the day of euthanasia. Morphology of apoptotic cells and macrophages undergoing efferocytosis were analyzed under an optical microscope (B and C, D and E respectively). N = 6. Results are representative of two independent experiments. Statistical analysis was done using two-tailed unpaired T test. P values were shown.

The activation of apoptosis in neutrophils followed by their efferocytosis by alternatively activated macrophages are key to immune cell clearance which contributes to the return to homeostasis^40^. Therefore, aiming to determine if AT-01-KG treatment induces apoptosis and efferocytosis, we first tested that hypothesis *in vitro*. BMDMs were infected with 0.1 MOI of HIN1 for 30min then treated with vehicle or 1nM of AT-01-KG for 24h. CFSE labeled apoptotic cells were then added for 1 hour in order to analyze efferocytosis by flow cytometry measuring F480^+^/CFSE^+^ cells. Treated cells presented a high frequency of macrophage efferocytosis compared to untreated cells (Fig. 4A). *In vivo*, treated mice also presented increased frequency of apoptotic cells (Fig. 4B and C) and macrophage efferocytosis (Fig. 4D and E) in the BALF, as assessed by morphology.

### 3.4. Levels of pro-inflammatory cytokines are decreased in the lungs of LXA_4_-treated mice

**Figure 5:**
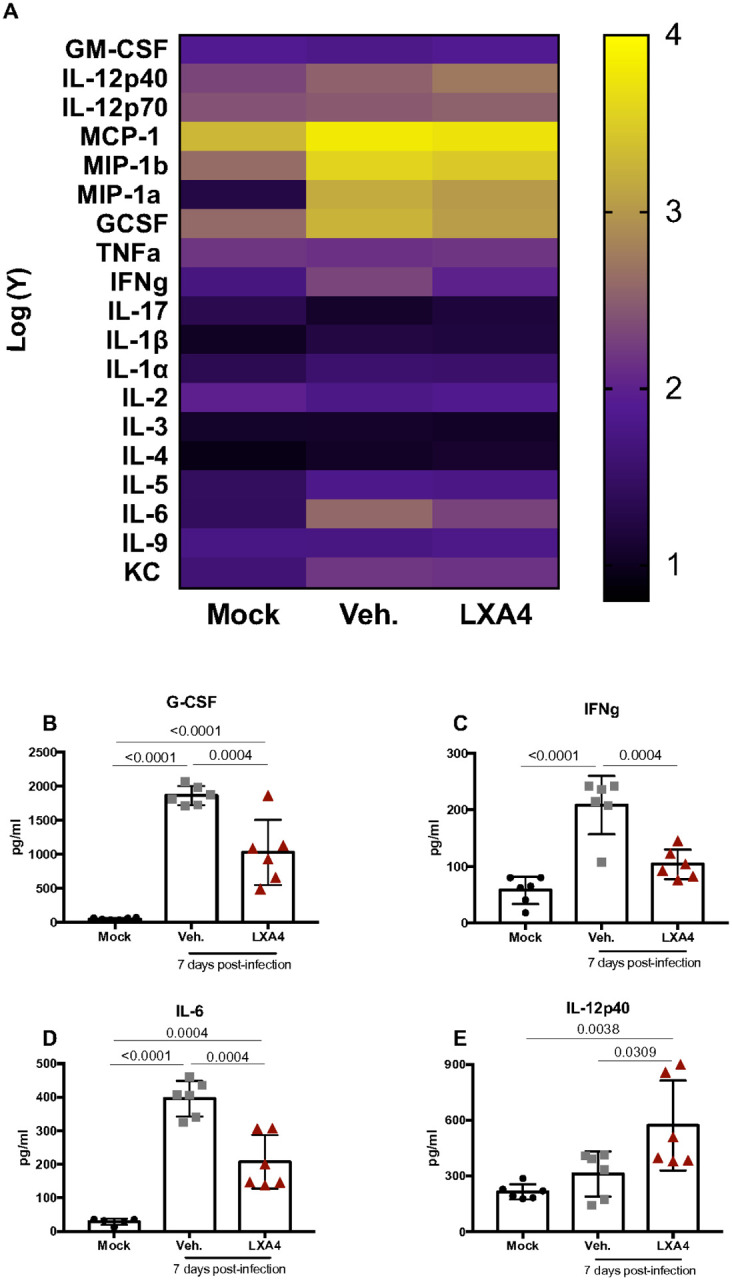
Lipoxin A_4_ treatment modulates cytokine profile. WT (C57Bl/6) mice were infected with Influenza A H1N1 and euthanized 7 days post infection. Mice were daily treated with vehicle (Veh.) or with lipoxin A4 (LXA4) from day 3 up to the day 6. Heat map (A), G-CSF (B), IFNγ (C), IL-6(D), IL-12p40 (E) were given by Luminex assay. N = 6. Results are representative of two independent experiments. Statistical analysis was done using One-way ANOVA, Turkey’s multiple comparisons test. P values were shown.

Since persistent cytokine production is associated with greater tissue damage and mortality^41^ and LXA_4_ has been shown to control levels of pro-inflammatory cytokine^42^ we aimed to assess the cytokine profile in the lungs of the mice through a Bio-plex 23-plex assay. A heat map (Fig. 5A) of all cytokines measured showed different cytokine profiles in the lungs of mice for each condition. Pro-inflammatory mediators such as G-CSF, IFNγ and IL-6 increases susceptibility to IAV lung injury^43^ and contributes to elevated neutrophil in ux^44^. We showed that treatment with LXA_4_ was able to decrease levels of those mediators (Figs. 5B–D). Interestingly, treated mice had increased levels of IL-12p40 in the lungs.

### 3.5. Treatment with LXA_4_ increased anti-inflammatory and regulatory T cells

**Figure 6:**
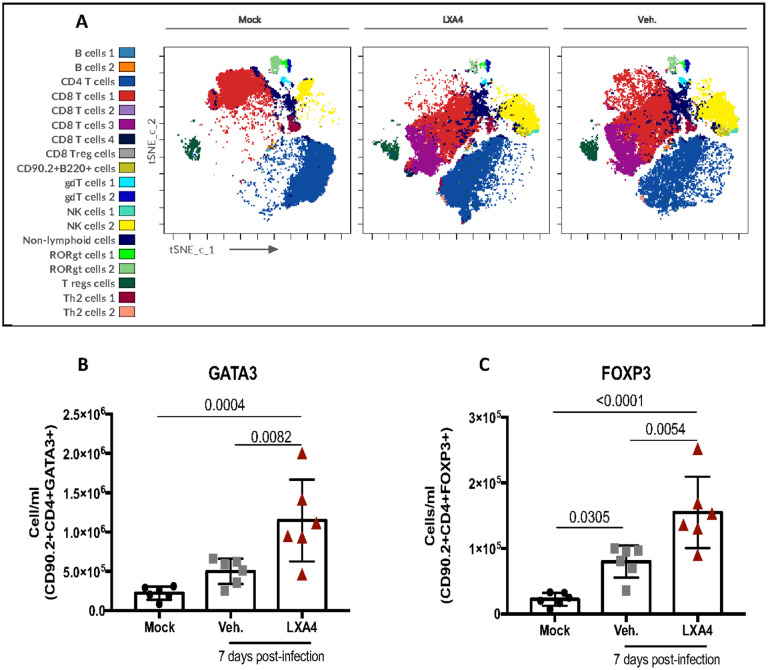
Lipoxin A_4_ treatment modulates lymphoid cells. WT (C57Bl/6) mice were infected with Influenza A H1N1 and euthanized 7 days post infection. Mice were daily treated with vehicle (Veh.) or with lipoxin A4 (LXA4) from day 3 up to the day 6. Dimensionality reduction analysis was performed using Cytobank on CD45+ cells(A) and on CD45+CD90.2+ cells(B) using t-SNE analysis and FlowSOM clustering to identify cell populations (left).Th2 cells (CD90.2+CD4+GATA3+)(B) and Tregs (CD90.2+CD4+FOXP3+)(C) were identified by traditional flow cytometry gatering strategies using FlowJo. N= 6. Results are representative of two independent experiments. Statistical analysis was done using or One-way ANOVA, Turkey’s multiple comparisons test. P values were shown.

Lymphoid cells play an important role controlling IAV infection, but they are also related to increased inflammatory response and damage^45,46^. Dimensionality reduction t-SNE analysis followed by FlowSOM clustering analysis was performed using Cytobank in order to characterize the lymphoid populations in the lungs of mice. Diverse cell clusters were generated in each condition. Clusters of CD8^+^ and CD4^+^ T cells along with clusters of NK cells were increased in the lungs of infected mice, especially in the lungs of non-treated mice (Fig. 6A). Traditional flow cytometry gating using FlowJo showed significantly increased numbers of T cells expressing GATA3 (anti-inflammatory T cells – Th2) (Fig. 6B) and T cells expressing FOXP3 (regulatory T cells) (Fig. 6C) in the lungs of treated mice compared to untreated mice.

### 3.6. AT-01-KG protection is FPR2/3-dependent

**Figure 7:**
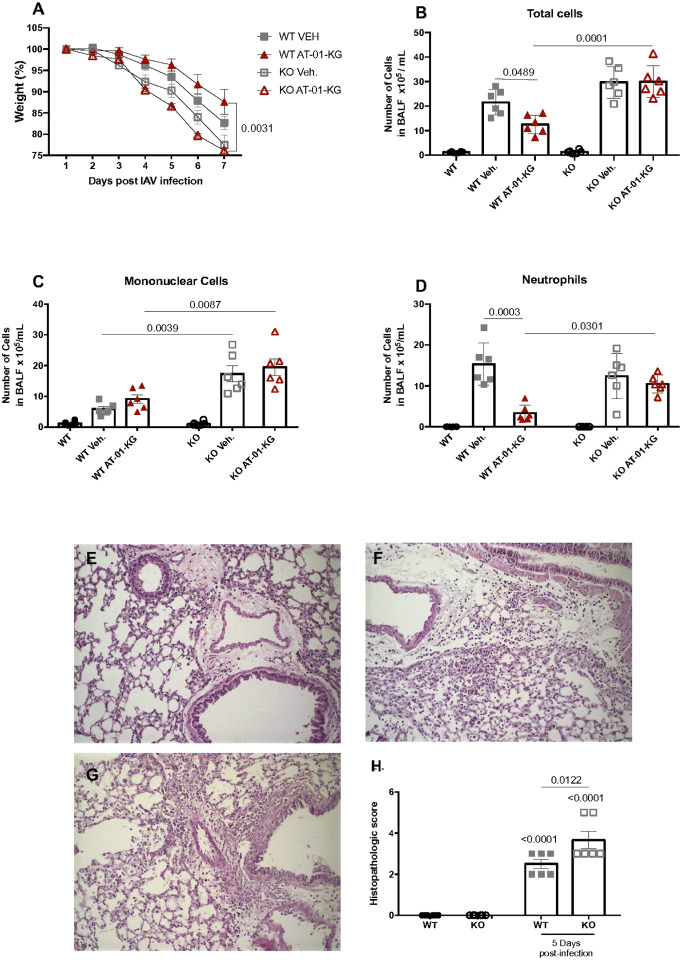
AT-01-KG protection is FPR2/3-dependent. WT (C57Bl/6) and FPR2/3 deficient (KO) mice were infected with Influenza A H1N1 and euthanized 5 days post infection. Mice were daily treated with vehicle (Veh.) or with lipoxin A_4_ analogous (AT-01-KG) from day 3 up to the day of euthanasia. Weight lost (A), BALF total cells, mononuclear cells and neutrophils (B, C and D respectively). Lung histology from mock mice, wild type and KO mice (E, F and G respectively) and histopathologic score (H) were performed. N = 6. Results are representative of two independent experiments. Statistical analysis was done using area under the curve or One-way ANOVA, Turkey’s multiple comparisons test. P values were shown.

Lipoxin A_4_ binds to FPR2 (FPR2/3 in mice), a member of the formyl peptide receptors (FPRs), triggering and an anti-inflammatory and pro-resolutive response^47^. In order to evaluate if the protection showed by the AT-01-KG treatment was via a direct effect, we infected FPR2/3^−/−^ mice (KO) with IAV and groups were assigned as mentioned before. We showed that KO treated mice had greater weight lost compared to the WT treated mice and no difference was seen when compared to KO vehicle group. (Fig. 7A). In addition, decreased cell infiltration induced by AT-01-KG treatment was no longer observed in the BALF of KO treated mice (Figs. 7B–D). Interestingly, the absence of FPR2/3 increased mononuclear cell infiltration (Fig. 7C) and tissue inflammation (Figs. 7E–G) as showed by the inflammatory histopathologic score (Fig. 7H).

## 4. DISCUSSION

Lipoxin A_4_ is one of the four forms of lipoxins first described in the 80’s^48^. It is able to improve inflammatory lung diseases^49,50^ and has an important role in tissue regeneration and homeostasis^51^.

The activation of the lipoxin pathway blocks the production of reactive oxygen species (ROS)^52^, which are associated with tissue damage in IAV infection^11^. Moreover, in 2020, Wang et al. showed that in a model of ventilator induced-lung injury, rats treated with LXA_4_ presented improved capillary permeability and reduction in tissue damage^53^. Our results align with those mentioned above since we showed that treatment with LXA_4_ reduced pulmonary damage caused by IAV infection. A therapeutic candidate able to decrease lung damage during this infection is highly relevant due to the fact that patients with IAV-induced acute respiratory failure require ventilatory support and the mechanical ventilation itself exacerbate the pulmonary damage impairing the survival rate^54^.

Tissue damage and impaired survival rate are caused by excessive cell recruitment leading to a persistent ongoing inflammation^55^. Recruited cells are responsible for the production of pro-inflammatory cytokines important for virus clearance, which leads to infection and inflammation control^56,57^. However, lung inflammatory responses can be a double-edged sword since it is associated with greater tissue damage and mortality^58^. In that regard, the production of anti-inflammatory and pro-resolving mediators is crucial to counter-balance the pro-inflammatory environment induced by the infection^59,60^. Here, the treatment with LXA_4_ reduced cell recruitment to the BAL of treated mice consistent with other studies showing that LXA_A_ blocks inflammatory cell migration^61,62^

The reduction of cell recruitment along with activation of neutrophil apoptosis and their efferocytosis by alternatively activated macrophages are crucial steps to switch on the resolution phase and prepare the tissue to return to homeostasis^52,63^. It has been demonstrated that LXA_4_ is able to enhance the resolution of inflammation through the activation of apoptotic pathways in neutrophils^64^ but inhibiting apoptosis in macrophages allowing the clearance of apoptotic cells in the tissue^65^. Efferocytosis is the clearance of apoptotic cells by macrophages which is followed by macrophage reprogramming towards an alternatively activated stage^66^ when superficial and internal anti-inflammatory cell makers such as CD206 and Arg1 are overexpressed^67,68^. Some studies have demonstrated the association between increased survival rate of influenza infected mice and decreased numbers of classically activated macrophages, together with the increased expression of alternatively activated macrophages markers^69,70^. Here we demonstrated that treatment with LXA_4_ reduced the frequency of classically activated exudate and interstitial macrophages. Treatment also increased the frequency of alternatively activated interstitial macrophages. Altogether, our *in vitro* and *in vivo* data suggests that the ability of LXA_4_ to activate apoptosis and efferocytosis and to induce macrophage reprogramming appears to contribute to the better outcome observed in this model of IAV infection.

Severe cases of IAV infection are marked by exuberant cytokine production with persistent innate immune cell infiltration and an exuberant T cell response^71^. Serum samples of patients with influenza-associated pneumonia showed a positive correlation between poor prognosis and elevated levels of IL-6, IFN-γ and G-CSF^72^. In mice, the production of those cytokines was also correlated with persistent neutrophil survival and infiltration, along with increased tissue damage^44,73^. Here, we also confirmed that correlation, which was improved by the treatment with LXA_4_. We also showed that the treatment increased the frequency of anti-inflammatory T cells, Th2, and regulatory T cells, T_regs_. In fact, the modulation of the adaptive immune response towards those subsets is associated with inhibition of neutrophil recruitment and better survival^74^. In addition, LXA_4_ induced increased frequency of macrophages which can be explained by higher levels of IL-12p40 in the lungs of treated mice since IL-12p40 induces macrophage in ltration^75,76^.

The anti-inflammatory and pro-resolutive effect of both LXA_4_ and FPR2 have been investigated not only in IAV infection, but also in other models of infection. Zhu, X. et al showed inhibition of pro-inflammatory mediators by LXA_4_ treatment in an *Aspergillus fumigatus* keratitis mouse model and that anti-inflammatory effect was reversed by the blockage of FPR2^77^. In 2017, another study showed that mice co-infected with IAV and *Streptococcus pneumoniae* presented an increase in neutrophil recruitment and neutrophil activation along with increased levels of proteins in BALF. Co-infected mice were then treated with resolving D1 - aspirin-triggered resolvin D1 (At-RvD1), another pro-resolving mediator that binds to FPR2. The treatment reduced lung damage and elastase activity as well as recruited neutrophils and levels of myeloperoxidase. Interestingly, FPR2 expression was elevated in the lungs of those mice^78^. Additionally, Schloer et al. showed the protective effect of the annexin A1/FPR2 pathway in a model of IAV infection. They showed that mice previously treated with annexin A1 and infected with IAV PR8 presented increased survival and decreased levels of IL-1β, IL-6 e MCP-1 on the third day post infection if compared with non-treated mice. The treatment also decreased lung damage and increased alveolar macrophages through FPR2 activation^79^. Here, our results indicate that, indeed, LXA_4_/FPR2 pathway has a protective role in a model of IAV infection since the protection induced by the treatment was reversed by the absence of FPR2/3. It is of interest to note that in FPR2/3−/− mice greater damage is observed compared to wild type mice. These observations are analogous to exacerbations of disease in models of arthritis and polymicrobial sepsis in FPR2/3^−/−^ mice highlighting the critical role of this receptor in immune homeostasis^80,81^.

## 5. CONCLUSION

Altogether, our results suggest that treatment with a LXA_4_ analogue post-IAV infection has a promising therapeutic effect. The effects of the compound are FPR2-dependent and work by inducing resolution of pulmonary inflammation, generating a regulatory phenotype in T cells and macrophages and decreasing tissue damage and mortality.

## References

[1] Hutchinson E. C. & Fodor E. Transport of the influenza virus genome from nucleus to nucleus. Viruses 5, 2424–46 (2013). 24104053 10.3390/v5102424 PMC3814596

[2] Akin L. & Gözel M. G. Understanding dynamics of pandemics. Turkish J. Med. Sci. 50, 515–519 (2020). 10.3906/sag-2004-133 PMC7195986 32299204

[3] Tavares L. P., Teixeira M. M. & Garcia C. C. The inflammatory response triggered by Influenza virus: a two edged sword. Inflamm. Res. 66, 283–302 (2017). 27744631 10.1007/s00011-016-0996-0

[4] WHO. Influenza (Seasonal). Available at: https://www.who.int/news-room/fact-sheets/detail/influenza-(seasonal).

[5] Batool S., Chokkakula S. & Song M.-S. Influenza Treatment: Limitations of Antiviral Therapy and Advantages of Drug Combination Therapy. Microorganisms 11, (2023). 10.3390/microorganisms11010183 PMC9865513 36677475

[6] Grousd J. A., Rich H. E. & Alcorn J. F. Host-Pathogen Interactions in Gram-Positive Bacterial Pneumonia. Clin. Microbiol. Rev. 32, (2019). 10.1128/CMR.00107-18 PMC6589866 31142498

[7] Edinger T. O., Pohl M. O. & Stertz S. Entry of influenza A virus: host factors and antiviral targets. J. Gen. Virol. 95, 263–277 (2014). 24225499 10.1099/vir.0.059477-0

[8] Nogusa S. RIPK3 Activates Parallel Pathways of MLKL-Driven Necroptosis and FADD-Mediated Apoptosis to Protect against Influenza A Virus. Cell Host Microbe 20, 13–24 (2016). 27321907 10.1016/j.chom.2016.05.011 PMC5026823

[9] Zhang T. Influenza Virus Z-RNAs Induce ZBP1-Mediated Necroptosis. Cell 180, 1115–1129.e13 (2020). 32200799 10.1016/j.cell.2020.02.050 PMC7153753

[10] Shim J. M., Kim J., Tenson T., Min J.-Y. & Kainov D. E. Influenza Virus Infection, Interferon Response, Viral Counter-Response, and Apoptosis. Viruses 9, (2017). 10.3390/v9080223 PMC5580480 28805681

[11] Liu H. Berberine suppresses influenza virus‐triggered NLRP3 in ammasome activation in macrophages by inducing mitophagy and decreasing mitochondrial ROS. J. Leukoc. Biol. 108, 253–266 (2020). 32272506 10.1002/JLB.3MA0320-358RR

[12] Julkunen I. Inflammatory responses in influenza A virus infection. Vaccine 19, S32–S37 (2000). 11163460 10.1016/s0264-410x(00)00275-9

[13] Gill M. A. Enhanced plasmacytoid dendritic cell antiviral responses after omalizumab. J. Allergy Clin. Immunol. 141, 1735–1743.e9 (2018). 28870461 10.1016/j.jaci.2017.07.035 PMC6013066

[14] Narasaraju T. Excessive neutrophils and neutrophil extracellular traps contribute to acute lung injury of influenza pneumonitis. Am. J. Pathol. 179, 199–210 (2011). 21703402 10.1016/j.ajpath.2011.03.013 PMC3123873

[15] Tate M. D. Neutrophils ameliorate lung injury and the development of severe disease during influenza infection. J. Immunol. 183, 7441–50 (2009). 19917678 10.4049/jimmunol.0902497

[16] Li K., McCaw J. M. & Cao P. Modelling within-host macrophage dynamics in influenza virus infection. J. Theor. Biol. 508, 110492 (2021). 32966828 10.1016/j.jtbi.2020.110492

[17] Chan L. L. Y. Host DNA released by NETosis in neutrophils exposed to seasonal H1N1 and highly pathogenic H5N1 influenza viruses. Respir. Res. 21, 160 (2020). 32576265 10.1186/s12931-020-01425-w PMC7310290

[18] Serhan C. N. & Levy B. D. Resolvins in inflammation: emergence of the pro-resolving superfamily of mediators. J. Clin. Invest. 128, 2657–2669 (2018). 29757195 10.1172/JCI97943 PMC6025982

[19] Costa V. V. Targeting the Annexin A1-FPR2/ALX pathway for host-directed therapy in dengue disease. Elife 11, (2022). 10.7554/eLife.73853 PMC8959599 35293862

[20] de Araújo S. Annexin A1-FPR2/ALX Signaling Axis Regulates Acute Inflammation during Chikungunya Virus Infection. Cells 11, (2022). 10.3390/cells11172717 PMC9454528 36078125

[21] Melo E. M. Relevance of angiotensin-(1–7) and its receptor Mas in pneumonia caused by influenza virus and post-influenza pneumococcal infection. Pharmacol. Res. 163, 105292 (2021). 33171305 10.1016/j.phrs.2020.105292

[22] Costa V. V., Resende F., Melo E. M. & Teixeira M. M. Resolution pharmacology and the treatment of infectious diseases. Br. J. Pharmacol. 181, 917–937 (2024). 38355144 10.1111/bph.16323

[23] Serhan C. N., Chiang N. & Van Dyke T. E. Resolving inflammation: dual anti-inflammatory and pro-resolution lipid mediators. Nat. Rev. Immunol. 8, 349–61 (2008). 18437155 10.1038/nri2294 PMC2744593

[24] Andrews D. & Godson C. Lipoxins and synthetic lipoxin mimetics: Therapeutic potential in renal diseases. Biochim. Biophys. Acta - Mol. Cell Biol. Lipids 1866, 158940 (2021). 10.1016/j.bbalip.2021.158940 33839296

[25] Galvão I. Therapeutic potential of the FPR2/ALX agonist AT-01-KG in the resolution of articular inflammation. Pharmacol. Res. 165, 105445 (2021). 33493655 10.1016/j.phrs.2021.105445

[26] Garcia C. C. Platelet-activating factor receptor plays a role in lung injury and death caused by Influenza A inflmice. PLoS Pathog. 6, e1001171 (2010). 21079759 10.1371/journal.ppat.1001171 PMC2974216

[27] Robinson K. M. Influenza A exacerbates Staphylococcus aureus pneumonia by attenuating IL-1β production in mice. J. Immunol. 191, 5153–9 (2013). 24089191 10.4049/jimmunol.1301237 PMC3827735

[28] O’Sullivan Timothy P., † Aromatic Lipoxin A4 and Lipoxin B4 Analogues Display Potent Biological Activities. (2007). doi:10.1021/JM060270D 17960922

[29] Börgeson E. Lipoxin A _4_ and benzo-lipoxin A _4_ attenuate experimental renal fibrosis. FASEB J. 25, 2967–2979 (2011). 21628447 10.1096/fj.11-185017

[30] Brennan E. P. Lipoxins Protect Against Inflammation in Diabetes-Associated Atherosclerosis. Diabetes 67, 2657–2667 (2018). 30213823 10.2337/db17-1317

[31] Melo E. M. Relevance of angiotensin-(1–7) and its receptor Mas in pneumonia caused by influenza virus and post-influenza pneumococcal infection. Pharmacol. Res. 163, 105292 (2021). 33171305 10.1016/j.phrs.2020.105292

[32] R. F Effect of Preventive or Therapeutic Treatment With Angiotensin 1–7 in a Model of Bleomycin-Induced Lung Fibrosis in Mice. J. Leukoc. Biol. 106, (2019). 10.1002/JLB.MA1218-490RR 31256436

[33] Helft J. GM-CSF Mouse Bone Marrow Cultures Comprise a Heterogeneous Population of CD11c(+)MHCII(+) Macrophages and Dendritic Cells. Immunity 42, 1197–211 (2015). 26084029 10.1016/j.immuni.2015.05.018

[34] Felix F. B. Blocking the HGF-MET pathway induces resolution of neutrophilic inflammation by promoting neutrophil apoptosis and efferocytosis. Pharmacol. Res. 188, 106640 (2023). 36627004 10.1016/j.phrs.2022.106640

[35] Magalhaes G. S. Angiotensin-(1–7) Promotes Resolution of Eosinophilic Inflammation in an Experimental Model of Asthma. Front. Immunol. 9, 58 (2018). 29434591 10.3389/fimmu.2018.00058 PMC5797293

[36] Gopal R. STAT2 Signaling Regulates Macrophage Phenotype During Influenza and Bacterial Super-Infection. Front. Immunol. 9, 2151 (2018). 30337919 10.3389/fimmu.2018.02151 PMC6178135

[37] Russo R. C. Phosphoinositide 3-kinase γ plays a critical role in bleomycin-induced pulmonary inflammation and fibrosis in mice. J. Leukoc. Biol. 89, 269–82 (2010). 21048214 10.1189/jlb.0610346

[38] Serhan C. N. Pro-resolving lipid mediators are leads for resolution physiology. Nature 510, 92–101 (2014). 24899309 10.1038/nature13479 PMC4263681

[39] Luo X. Macrophage Reprogramming via Targeted ROS Scavenging and COX-2 Downregulation for Alleviating Inflammation. Bioconjug. Chem. 34, 1316–1326 (2023). 37330989 10.1021/acs.bioconjchem.3c00239

[40] Serhan C. N. Pro-resolving lipid mediators are leads for resolution physiology. Nature 510, 92–101 (2014). 24899309 10.1038/nature13479 PMC4263681

[41] Verma A. K., Bauer C., Palani S., Metzger D. W. & Sun K. IFN-γ Drives TNF-α Hyperproduction and Lethal Lung Inflammation during Antibiotic Treatment of Postinfluenza Staphylococcus aureus Pneumonia. J. Immunol. 207, 1371–1376 (2021). 34380647 10.4049/jimmunol.2100328

[42] Liu X. Lipoxin A4 and its analog suppress inflammation by modulating HMGB1 translocation and expression in psoriasis. Sci. Rep. 7, 7100 (2017). 28769106 10.1038/s41598-017-07485-1 PMC5541073

[43] Califano D. IFN-γ increases susceptibility to influenza A infection through suppression of group II innate lymphoid cells. Mucosal Immunol. 11, 209–219 (2018). 28513592 10.1038/mi.2017.41 PMC5693789

[44] Dienz O. Essential role of IL-6 in protection against H1N1 influenza virus by promoting neutrophil survival in the lung. Mucosal Immunol. 5, 258–266 (2012). 22294047 10.1038/mi.2012.2 PMC3328598

[45] van de Sandt C. E. Human CD8+ T Cells Damage Noninfected Epithelial Cells during Influenza Virus Infection In Vitro. Am. J. Respir. Cell Mol. Biol. 57, 536–546 (2017). 28613916 10.1165/rcmb.2016-0377OC

[46] Frank K. & Paust S. Dynamic Natural Killer Cell and T Cell Responses to Influenza Infection. Front. Cell. Infect. Microbiol. 10, 425 (2020). 32974217 10.3389/fcimb.2020.00425 PMC7461885

[47] Lee C., Han J. & Jung Y. Formyl peptide receptor 2 is an emerging modulator of inflammation in the liver. Exp. Mol. Med. 55, 325–332 (2023). 36750693 10.1038/s12276-023-00941-1 PMC9981720

[48] Serhan C. N. & Samuelsson B. Lipoxins: a new series of eicosanoids (biosynthesis, stereochemistry, and biological activities). Adv. Exp. Med. Biol. 229, 1–14 (1988). 10.1007/978-1-4757-0937-7_1 3048058

[49] Li Y. Lipoxin A4 protects against paraquat-induced acute lung injury by inhibiting the TLR4/MyD88‐mediated activation of the NF-κB and PI3K/AKT pathways. Int. J. Mol. Med. 47, (2021). 10.3892/ijmm.2021.4919 PMC7992923 33760150

[50] Sekheri M., El Kebir D., Edner N. & Filep J. G. 15-Epi-LXA4 and 17-epi-RvD1 restore TLR9-mediated impaired neutrophil phagocytosis and accelerate resolution of lung inflammation. Proc. Natl. Acad. Sci. U. S. A. 117, 7971–7980 (2020). 32205444 10.1073/pnas.1920193117 PMC7149425

[51] Yang J. Lipoxin A4 ameliorates lipopolysaccharide-induced lung injury through stimulating epithelial proliferation, reducing epithelial cell apoptosis and inhibits epithelial–mesenchymal transition. Respir. Res. 20, 192 (2019). 31438948 10.1186/s12931-019-1158-z PMC6704532

[52] Serhan C. N. The Atlas of Inflammation Resolution (AIR). Mol. Aspects Med. 74, 100894 (2020). 32893032 10.1016/j.mam.2020.100894 PMC7733955

[53] Wang Q., Xu G.-X., Tai Q.-H. & Wang Y. Lipoxin A4 Reduces Ventilator-Induced Lung Injury in Rats with Large-Volume Mechanical Ventilation. Mediators Inflamm. 2020, 6705985 (2020). 10.1155/2020/6705985 PMC7704204 33299377

[54] Pimenta L. B. M. Protective mechanical ventilation in suspected influenza infection. Rev. Soc. Bras. Med. Trop. 53, e20190481 (2020). 33027412 10.1590/0037-8682-0481-2019 PMC7534969

[55] Laghlali G., Lawlor K. E. & Tate M. D. Die Another Way: Interplay between Influenza A Virus, Inflammation and Cell Death. Viruses 12, (2020). 10.3390/v12040401 PMC7232208 32260457

[56] Troisi E. M., Nguyen B. H., Baxter V. K. & Griffin D. E. Interferon regulatory factor 7 modulates virus clearance and immune responses to alphavirus encephalomyelitis. J. Virol. 97, e0095923 (2023). 37772825 10.1128/jvi.00959-23 PMC10617562

[57] Wang R. Influenza A virus protein PB1-F2 impairs innate immunity by inducing mitophagy. Autophagy 17, 496–511 (2021). 32013669 10.1080/15548627.2020.1725375 PMC8007153

[58] Aguilera E. R. & Lenz L. L. Inflammation as a Modulator of Host Susceptibility to Pulmonary Influenza, Pneumococcal, and Co-Infections. Front. Immunol. 11, 105 (2020). 32117259 10.3389/fimmu.2020.00105 PMC7026256

[59] Roquilly A. Local Modulation of Antigen-Presenting Cell Development after Resolution of Pneumonia Induces Long-Term Susceptibility to Secondary Infections. Immunity 47, 135–147.e5 (2017). 28723546 10.1016/j.immuni.2017.06.021

[60] West E. E. Loss of CD4+ T cell-intrinsic arginase 1 accelerates Th1 response kinetics and reduces lung pathology during influenza infection. Immunity 56, 2036–2053.e12 (2023). 37572656 10.1016/j.immuni.2023.07.014 PMC10576612

[61] Serhan C. N. Design of lipoxin A4 stable analogs that block transmigration and adhesion of human neutrophils. Biochemistry 34, 14609–15 (1995). 7578068 10.1021/bi00044a041

[62] Fu T. Therapeutic Potential of Lipoxin A 4 in Chronic Inflammation: Focus on Cardiometabolic Disease. ACS Pharmacol. Transl. Sci. 3, 43–55 (2020). 32259087 10.1021/acsptsci.9b00097 PMC7088989

[63] Vasconcelos D. P. Modulation of the inflammatory response to chitosan through M2 macrophage polarization using pro-resolution mediators. Biomaterials 37, 116–23 (2015). 25453942 10.1016/j.biomaterials.2014.10.035

[64] El Kebir D. & Filep J. G. Targeting neutrophil apoptosis for enhancing the resolution of inflammation. Cells 2, 330–48 (2013). 24709704 10.3390/cells2020330 PMC3972676

[65] Prieto P. Lipoxin A4 impairment of apoptotic signaling in macrophages: implication of the PI3K/Akt and the ERK/Nrf-2 defense pathways. Cell Death Differ. 17, 1179–88 (2010). 20094061 10.1038/cdd.2009.220

[66] Mehrotra P. & Ravichandran K. S. Drugging the efferocytosis process: concepts and opportunities. Nat. Rev. Drug Discov. 21, 601–620 (2022). 35650427 10.1038/s41573-022-00470-y PMC9157040

[67] Du Q. Transfusion of CD206+ M2 Macrophages Ameliorates Antibody-Mediated Glomerulonephritis in Mice. Am. J. Pathol. 186, 3176–3188 (2016). 27855848 10.1016/j.ajpath.2016.08.012

[68] Ishida K. Induction of unique macrophage subset by simultaneous stimulation with LPS and IL-4. Front. Immunol. 14, 1111729 (2023). 10.3389/fimmu.2023.1111729 PMC10167635 37180123

[69] Chida J. Prion protein signaling induces M2 macrophage polarization and protects from lethal influenza infection in mice. PLoS Pathog. 16, e1008823 (2020). 32845931 10.1371/journal.ppat.1008823 PMC7489546

[70] Halstead E. S. GM-CSF overexpression after influenza a virus infection prevents mortality and moderates M1-like airway monocyte/macrophage polarization. Respir. Res. 19, 3 (2018). 29304863 10.1186/s12931-017-0708-5 PMC5756339

[71] Kalil A. C. & Thomas P. G. Influenza virus-related critical illness: pathophysiology and epidemiology. Crit. Care 23, 258 (2019). 31324202 10.1186/s13054-019-2539-x PMC6642581

[72] Zhang J. Interleukin-6 and granulocyte colony-stimulating factor as predictors of the prognosis of influenza-associated pneumonia. BMC Infect. Dis. 22, 343 (2022). 35382755 10.1186/s12879-022-07321-6 PMC8983324

[73] Klomp M., Ghosh S., Mohammed S. & Khan M. N. From virus to inflammation, how influenza promotes lung damage. J. Leukoc. Biol. 110, 115–122 (2021). 32895987 10.1002/JLB.4RU0820-232R PMC7937770

[74] Herold S., Becker C., Ridge K. M. & Budinger G. R. S. Influenza virus-induced lung injury: pathogenesis and implications for treatment. Eur. Respir. J. 45, 1463–78 (2015). 25792631 10.1183/09031936.00186214

[75] Ha S. J. A Novel Function of IL-12p40 as a Chemotactic Molecule for Macrophages. J. Immunol. 163, 2902–2908 (1999). 10453037

[76] Cooper A. M. & Khader S. A. IL-12p40: an inherently agonistic cytokine. Trends Immunol. 28, 33–38 (2007). 17126601 10.1016/j.it.2006.11.002

[77] Zhu X. Lipoxin A4 activates ALX/FPR2 to attenuate inflammation in Aspergillus fumigatus keratitis. Int. Immunopharmacol. 96, 107785 (2021). 34162149 10.1016/j.intimp.2021.107785

[78] Wang H. Aspirin-triggered resolvin D1 reduces pneumococcal lung infection and inflammation in a viral and bacterial coinfection pneumonia model. Clin. Sci. (Lond). 131, 2347–2362 (2017). 28779028 10.1042/CS20171006

[79] Schloer S. The annexin A1/FPR2 signaling axis expands alveolar macrophages, limits viral replication, and attenuates pathogenesis in the murine influenza A virus infection model. FASEB J. 33, 12188–12199 (2019). 31398292 10.1096/fj.201901265R PMC6902725

[80] Dufton N. Anti-inflammatory role of the murine formyl-peptide receptor 2: ligand-specific effects on leukocyte responses and experimental inflammation. J. Immunol. 184, 2611–2619 (2010). 20107188 10.4049/jimmunol.0903526 PMC4256430

[81] Chen J. Formyl Peptide Receptor Type 2 Deficiency in Myeloid Cells Ampli es Sepsis-Induced Cardiac Dysfunction. J. Innate Immun. 15, 548–561 (2023). 37068475 10.1159/000530284 PMC10315071

